# Continuous Gum Work

**Published:** 1883-06

**Authors:** A. B. Verrier


ARTICLE II.

### CONTINUOUS GUM WORK.

BY A. B. VERRIER, L. D. S. I.

We are especially indebted to Dr. John Allen, a distinguished American Dentist, for very important and original improvements in the compounding and manufacturing of the materials employed in Continuous Gum Work. The body and the gum enamels, prepared according to Dr. Allen's formula, may be obtained at any of the leading Dental Depots. Basing my opinion upon success and experience in Continuous Gum Work, I am led to prefer the material as compounded by Dr. Allen; it being more uniform in its results, and, when properly manipulated, is certainly not so liable to fracture when worn in the mouth. I have encountered many difficulties in attempting to work successfully the Continuous Gum Process with the ordinary furnaces in use, principally on account of the great labor involved in their proper management, in consequence of which many have abandoned it and condemned one of the most beautiful processes in Mechanical Dentistry. The huge dimensions of former furnaces, the difficulty encountered in their management, the time occupied in firing and cooling the work, besides the annoyance caused by dirt and dust, and the risk of being roasted oneself, precluded their general adoption.

A denture made in Continuous Gum Work, when artistically put out of hand, and the base-plate scientifically adapted to the oral tissues, is certainly the most beautiful production of Dental prosthesis, besides being the purest and sweetest that can be worn in the mouth. The objection urged against Continuous Gum Work by many Dentists is its weight; but although somewhat heavier than ordinary work, it has many important advantages, viz., its natural appearance, and the very beatiful and natural effects produced in the thorough restoration of the lost tissues. Unless the work is thoroughly and well executed from the procuring of a perfect model of the dental arch to the completion of the denture, failure will be inevitable. To retain a denture successfully in contact with the tissues of the oral cavity, depends upon the accuracy of its adaption, and the consequent exclusion of the atmospheric air from between the gum and base-plate. If we could exhaust the air entirely from between the gum and plate so that the full force of the atmosphere be exerted upon its lingual surface, it would adhere with great tenacity; resisting a strain equal to fifteen pounds to each square inch of its surface. I have devoted time and attention to the construction of dental atmospheric plates, with varied success, but for some time past I have relied upon a style of plaster especially applicable to the Continuous Gum Process, possessing many advantages over the ordinary mode of construction; several of which I may as well mention. Its freedom from any tendency to warp in the process of firing the gum body or the enamels; thorough stability in the mouth; great rigidy under the pressure of mastication; perfect adhesion and freedom from any tendency to injure the mucous membrane; its comparitive lightness and simplicity of construction.

I always procure models of the mouth in plaster of Paris. Before casting the metal dies an air-chamber disc or shield, oval in form, must be moulded on the surface of the plaster model, and should not exceed in diameter half-an-inch, while the thickness should not be more than one-sixteenth. Having decided upon the dimensions of the base-plate, allow an extra width of one-sixteenth of an inch fuller all round, as the edge must be turned up and present an abrupt shoulder to the margin of the gum enamel. The base-plate may now be struck up to the required form, and the edge turned up with a suitable pair of pliers and hammer. Being satisfied with the fit of the plate, secure it to the plaster model with adhesive wax. A layer of very thin sheet bees' wax (not thicker than a threepenny-piece) is now to cover the whole lingual surface of the swaged metal plate to within one-eighth of an inch of its entire border, cutting away the wax over the plate covering the air-chamber, leaving the wax level with its surface. The edge of the wax-plate, still in position upon the model, should be nicely bevelled off at its outer border to a very thin layer. Mould this in sand and procure metal dies. A second plate is now to be struck up large enough to cover the surface of the wax-plate and air-chamber, allowing width enough for soldering. The two plates are now to be removed from the plaster cast, and thoroughly cleaned by boiling in a weak solution of sulphuric acid and water, washing and brushing with pumice powder in the lathe. Before uniting the two plates, a few very fine holes must be drilled through the side of the chamber struck up in the base or first plate, to prevent the bursting of the compound plate in the process of soldering, and also to admit of the exhaustion of the air from between the plates by the patient when worn in the mouth. The two plates are now to be united with pieces of fine gold around their margins, using the fine gold freely, as the platinum absorbs most of it in the after process of firing. If the union of the two plates is perfect, adhesion will be so complete when tried in the mouth as to require a very considerable force to displace it, the more so if the patient be requested to exhaust the air from between the plates by merely closing the mouth and gently sucking it. I do not advise this course, as the adhesion is so thorough as to require a very considerable pull to remove it, resulting from the pressure of the external atmosphere upon the whole outer area of the plate, induced by the partial vacum created between the plates, causing it to adhere with great tenacity. Being perfectly satisfied with the adaption of the base-plate, proceed to take the articulation in the usual way—attaching to the ridge of each plate a root of adhesive wax as near as possible in width to the length of the teeth required for each plate respectfully. Place the plates with the wax in position in the mouth, requesting the patient to throw the head well backwards, so that the lower jaw shall be held back by the tension of the muscles of the neck when the mouth is closed. Antagonize the wax rims and mark the position of the frænum at the median line; and then adjust the whole in the articulator. Having selected a suitable set of continuous-gum root-teeth, proceed to set them up in the wax rims upon the base-plate, beginning with the six front upper and lower teeth. Place the plate with the wax and teeth attached in the mouth, and arrange the teeth as to length, fulness, and general outline—avoiding artificiality. The whole set of root-tooth may now be set up in the wax rims, allowing their roots to rest upon the plate and finally tried into the mouth, adjusting any particular tooth or teeth according to your æsthetic taste and the requirements of the case; for on this stage of the work depends the success of the denture as to shade, size, length, fulness, outline, and natural appearance when finished.

The adhesive wax adherent to the labial surfaces of the teeth must next be removed with a very sharp instrument, leaving the front of the teeth well exposed, so that they shall be held securely by the investient during the process of backing. The labial surfaces and cutting edges of the teeth are now to be covered with a very thin coating of fine clean plaster of Paris, and allowed to set firmly. After which, another layer an inch thick, composed of equal quantities of finely powdered asbestos and plaster of Paris in water, is placed around the outside of the previous covering and the plate. The best way is to turn the batter out upon a slab, previously laying a few folds of blotting paper on it to remove any excess of moisture, and then press the plate with the teeth upwards into the mixture to within half-an-inch of the slab. Then with a spatula or suitable knife bring the investiment up around and over the previous coating of plaster. The investient should be sufficiently thick to hold the teeth in position during the process of fitting and securing the platinum backs; when set, the remaining wax adherent to the teeth and plate can be removed with boiling water. The pins at the back of the teeth are now to be bent out, and a rim of soft platinum, No. 6 gauge fitted to the backs of the teeth and base-plate, bending down the pins to secure the platinum backing in place, which may be put on in several strips, with the ends overlapping each other; turning over the lower edge of the backing in positive contact with and upon the base-plate and should be one continuous band all around when soldered. Pieces of fine gold moistened with ground borax and water are to be put upon the parts to be soldered.

The work is now ready for soldering, which I always do in the furnace, as follows :—Heat the furnace to a good red heat, and then turn off the gas supply. The work may now be put upon a plain slab, and introduced into the furnace to dry thoroughly, drawing the work forward to the furnace door, throw a lighed match into the furnace, and turn the gas full on, at the same time working the foot-blower. Heat up the furnace a second time gradually, introducing the work to be soldered ; and apply the heat until the process of soldering is complete. The work must be allowed to cool slowly before removing the investient. The piece may now be well washed in water, using a stiff brush, and afterwards boiled out in a weak solution of sulphuric acid and water, and then washed again to remove any trace of the acid. Now roughen the plate with a sharp graver to secure the gum body to the plate.

The piece is now ready to receive the first application of the ceramic paste or gum body. For this purpose, I have two shallow earthenware pots with good fitting covers to contain the gum body and the enamel; one or two thin well-tempered spatulas ; a suitable agate burnisher for compressing the compound; also one or two fine camel-hair brushes for laying on the paste, and one rather stiffer for removing any grit adherent to the surfaces of the teeth previous to firing. Also three wide-mouthed stoppered bottles containing distilled water for washing the brushes during the process of laying on the gum body and enamel. Before commencing to lay on thc ceramic paste, see that everything is perfectly clean, and the floor of the laboratory is well sprinkled with water, to prevent dust rising and so falling upon the work. A clean sheet of blotting paper should be laid upon the bench or table on which the work and necessary articles used in the process can be put. Holding the work in the fingers of the left hand which shculd be perfectly clean, proceed to lay on ceramic paste.

Mix with distilled water in one of the earthenware pots sufficient gum body for the case in hand, to the consistency of thick cream. With spatula and camel-hair brushes proceed to lay on the paste, working it well into the spaces between, under and around the teeth; removing any excess of moisture with small pieces of blotting paper; and then thoroughly condensing it with the agate burnisher, covering the surface of the plate to about the thickness of coarse paper. The layer of paste is then carved to represent the gum, palatal arch and rugæ; taking particular care to keep the necks of the teeth well defined; and finally, before firing the work, the surfaces of the teeth should be freed from grit or particles of gum body by brushing them with a moderately stiff camel-hair brush. The work is now ready for firing, but before applying the whole volume of heat the piece should be slowly but thoroughly dried, by turning on the gas and drying it at the furnace door, introducing the muffle containing the work gradually. The work should rest upon a piece of platinum wire, bent so as to form three supports, and should be placed just inside the mouth of the muffle. When the work is thoroughly annealed, throw a lighted match into the furnace, and turn the gas fully on, at the same time working the foot-blower, gradually introducing the muffle as the heat is increased, until the body becomes semi-vitrified, which is sufficient for the first firing. To those unacquainted with the firing of continuous gum work I would advise, in commencing the process, to use what is termed a test-piece, which is simply a piece of platinum wire about six inches long, flattened at one end, on which is laid a little of the gum body or enamel. This may be removed from the furnace at intervals of a few minutes and examined. The gas and air is then turned off and the furnace allowed to cool until the red heat has disappeared from the muffle, which may then be withdrawn, and the work allowed to cool in the muffle at the furnace door. The work is then ready for a second application of the gum body, for the purpose of repairing any defects due to the shrinkage caused by the first firing. This being done, the piece is again fired, allowing the second bake to be a little harder than the first, but not so much as to appear glossy. The muffle may now be removed from the furnace, and the work allowed to cool as before.

The denture is now ready to receive an application of the gum enamel, which is mixed in the same manner as the gum body in an earthenware pot, or other suitable vessel. A thin layer is then to be put on with spatula and suitable camel-hair brushes, covering the whole surface of the body, varying the thickness so as to represent the different shades to be observed in the natural gum. The crowns of the teeth must be well marked, the gum well defined, and the rugæ of the palate well moulded and prominent, removing any superfluous moisture with small pieces of blotting paper. Great care should be taken to remove with dry brushes all particles of the gum body and enamel, or any other substance adherent to the crowns of the teeth previous to firing. Having carefully put on the enamel, the work is then ready for the final baking or firing, which should be conducted as advised for firing the body, excepting that the heat must be stronger, in order to produce a moderately glossy appearance of the enamel when fused. The muffle may be removed from the furnace, and the work allowed to cool. It is advisable not to remove the work from the muffle until it is quite cold.

The piece is now to be boiled out in a very weak solution of sulphuric acid and water, preparatory to finishing the platinum plate in the usual way, by filing, sand-papering, stoning, etc., and the final gilding process. This latter process may be simply done in the following way:—Take 20 grains of waste gold stopping, and put into a shallow evaporating dish with about 2 drachms of nitro-muriatic acid, and evaporate nearly to dryness over a heated sand bath. Dissolve 6 drachms of cyanide of potassium in 24 ounces of distilled or rain water, and then add the chloride of gold. This is the gilding solution. The plate to be gilded should be well cleaned with powdered pumice stone in the lathe, and thoroughly washed in clean water. To gild the work, put it into a suitable vessel with sufficient gilding solution to cover the article, gently warming it over a sand bath. A strip of clean sheet zinc put into the solution, and in contact with the plate, will complete the gilding process, and give a very fine and rich coating of gold equal, if not superior, to gilding by the aid of a battery. The edges of the plate should finally be burnished with the agate burnisher and plenty of soapy water.—*London Dental Record.*

